# Gate-Stack Engineering to Improve the Performance of 28 nm Low-Power High-K/Metal-Gate Device

**DOI:** 10.3390/mi12080886

**Published:** 2021-07-27

**Authors:** Jeewon Park, Wansu Jang, Changhwan Shin

**Affiliations:** 1 Department of Semiconductor and Display Engineering, Sungkyunkwan University, Suwon 16419, Korea; tmfktys@g.skku.edu; 2 Foundry, Samsung Electronics, Yongin 17113, Korea; wansu.jang@samsung.com; 3 Department of Electrical and Computer Engineering, Sungkyunkwan University, Suwon 16419, Korea

**Keywords:** high-k/metal-gate, HfSiON, HfSiO, gate-stack engineering

## Abstract

In this study, a gate-stack engineering technique is proposed as a means of improving the performance of a 28 nm low-power (LP) high-k/metal-gate (HK/MG) device. In detail, it was experimentally verified that HfSiO thin films can replace HfSiON congeners, where the latter are known to have a good thermal budget and/or electrical characteristics, to boost the device performance under a limited thermal budget. TiN engineering for the gate-stack in the 28 nm LP HK/MG device was used to suppress the gate leakage current. Using the proposed fabrication method, the on/off current ratio (*I*_on_/*I*_off_) was improved for a given target *I*_on_, and the gate leakage current was appropriately suppressed. Comparing the process-of-record device against the 28 nm LP HK/MG device, the thickness of the electrical oxide layer in the new device was reduced by 3.1% in the case of n-type field effect transistors and by 10% for p-type field effect transistors. In addition, the reliability (e.g., bias temperature instability, hot carrier injury, and time-dependent dielectric breakdown) of the new device was evaluated, and it was observed that there was no conspicuous risk. Therefore, the HfSiO film can afford reliable performance enhancement when employed in the 28 nm LP HK/MG device with a limited thermal budget.

## 1. Introduction

To meet the needs of a hyper-connected society, the need for various types of integrated circuit (IC) chips has dramatically increased. Although fin-shaped field-effect transistors (FinFETs) have been widely adopted in low-power/high-performance IC chips over the last a few decades, they are still not very cost-effective for some applications. If a technique for dramatically improving the performance of planar bulk transistors in legacy technology (e.g., 28 nm technology) can be developed, it would potentially replace cutting-edge FinFET devices.

To improve the performance of 28 nm gate-first high-k/metal-gate (HK/MG) devices, it is essential to optimize the quality of high-k films. In terms of thermal budget, the quality of HfSiON films is known to be better than that of HfSiO congeners [1,2,3,4,5,6]. For this reason, HfSiON is generally preferred because the gate stack in the gate-first process is more likely to be exposed to heat and/or stress. Nitride in HfSiON is known to prevent crystallization, as well as to improve the film properties. However, studies have shown that it also has a negative impact, e.g., mobility degradation due to nitrides [7]. If HfSiO film can be optimized to minimize the mobility degradation as well as to ensure reliability, better device performance can be achieved. Atomic layer deposition (ALD) is widely used for HfSiO deposition, and it can ensure a better film quality in a hydrophilic environment [8,9,10].

The gate leakage current is a critical factor that directly impacts performance characteristics [11]. As shown in [12], efforts to suppress leakage current even in the latest transistor structures have been continuously studied. TiN engineering (e.g., by controlling the thickness of TiN) was carried out to suppress the gate leakage current. However, the unstable membranes of the thin TiN layer would be likely to increase the gate leakage current, and, thus, a thick TiN is thought to be a better choice in preventing the formation of interfacial traps, such as oxygen void diffusion and boron penetration [13]. In particular, it improves the threshold voltage (*V*_th_) distribution of p-type field-effect transistors (PFETs), and it is known to effectively improve the reliability, such as the bias temperature instability (BTI) [14].

## 2. Materials and Methods

**Figure 1:**
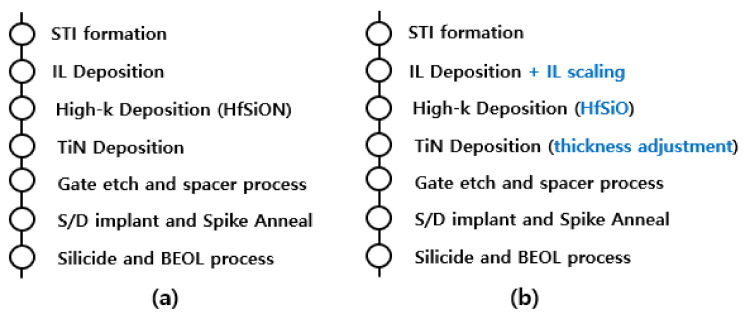
Fabrication process flow of (**a**) process-of-record (POR) and (**b**) the one proposed in this study.

In this study, a metal oxide semiconductor (MOS) structure was fabricated to develop a gate-first high-k/metal-gate 28 nm LP transistor on an epitaxial Si (100) wafer. In the case of the process-of-record (POR) MOS structure, SiO_2_/HfSiON/TiN was used as an interlayer (IL), high-k material, and metal-gate, respectively, and they were deposited in order. For the newly proposed fabrication process, the interlayer (IL) of SiO_2_ was used, and, then, cleaning steps for scaling down the IL layer were added to maximize the device performance. IL scaling also aims to create a hydrophilic environment for the ALD process. However, excessive IL scaling affects the reliability of oxide film quality and cannot be performed in large quantities. As the IL layer was very thin, the physical measurement was hard to use, and, thus, an electrical measurement was used to monitor the IL layer thickness. When depositing HfSiO instead of HfSiON, ALD was used. This was conducted with the intention of avoiding the degradation effect of nitride. Additionally, the TiN thickness was increased by 15% in the developed recipe to suppress the gate leakage current. This was to compensate for leakage caused by the decreased IL layer. Afterwards, gate etching, spacer formation, source/drain implantation, spike annealing, and silicide processes were carried out in both fabrication procedures. Figure 1 summarizes the process flow charts.

The performance improvement was confirmed by comparing the value of *I_on_/I_off_* against the gate leakage current and electrical oxide thickness (EOT or *T_ox_*). *T_ox_* was quantitatively estimated based on the measured capacitance in the inversion mode of MOS device. The capacitance characteristics can be inferred from the changes in EOT. Note that the gate leakage current indicates the gate current under *V*_GS_ = *V*_DD_ (=1 V in this study) and *V*_DS_ = 0 V, for the n-type field-effect transistor (NFET). *I_on_* is the measured current under *V*_G_ = *V*_D_ = *V*_DD_ (=1 V in this study), and *I_off_* is the measured leakage current under *V*_G_ = 0 V and *V*_D_ = *V*_DD_ (=1 V in this study). All electrical data were measured at room temperature. All samples were analyzed under the same conditions. The test pattern used for verification was designed with a 28 nm gate length, and the ratio of n-type MOSFET’s gate width to p-type’s one, i.e., *W*_n-type_:*W*_p-type_ = 1:1.4. The gate leakage current and *T_ox_* were measured in a large-scale pattern to better analyze the interfacial properties. Note that thirteen samples per group were used for evaluation. The geometrical parameters of device structure used in this work are summarized in Table 1.

## 3. Results and Discussion

**Figure 2:**
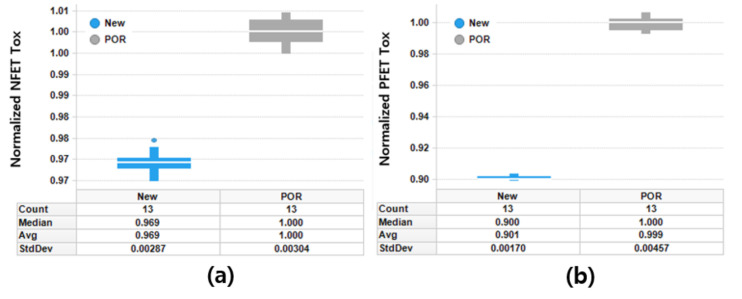
Normalized electrical oxide thickness of (**a**) n-type field-effect transistor (NFET) and (**b**) p-type field-effect transistor (PFET).

**Figure 3:**
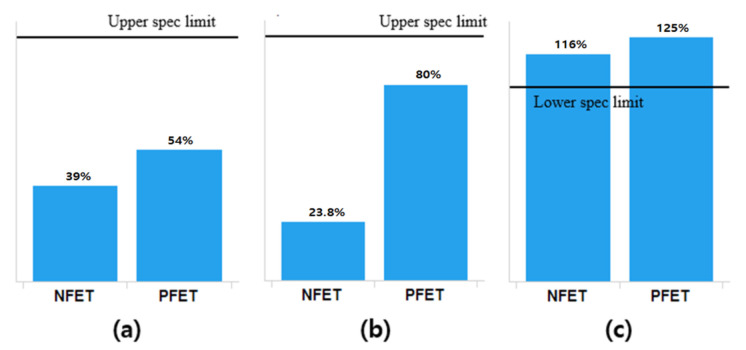
Reliability test result: (**a**) hot-carrier injection (HCI), (**b**) bias temperature instability (BTI), and (**c**) time-dependent dielectric breakdown (TDDB).

The thickness of the IL layer in the process-of-record (POR) samples is different from that in the newly fabricated samples because of the scaling down of the IL layer in the new samples. Thus, the electrical oxide thickness was measured for comparative purposes. With the newly proposed fabrication process, the gate leakage of MOSFET was well suppressed. Compared to the POR device, the electrical oxide thickness in the new device was reduced by 3.1% for the n-type field-effect transistor (NFET) and 10% for the PFET (see Figure 2). It is clear that the thinner the gate oxide, the better the device performance of MOSFET. However, the limit for reducing the physical oxide thickness should be set/determined by reliability requirements. The reliability of the samples fabricated by the newly proposed process flow was evaluated in terms of hot-carrier injection (HCI), bias temperature instability (BTI), and time-dependent dielectric breakdown (TDDB). The measured results are summarized in Figure 3.

**Figure 4:**
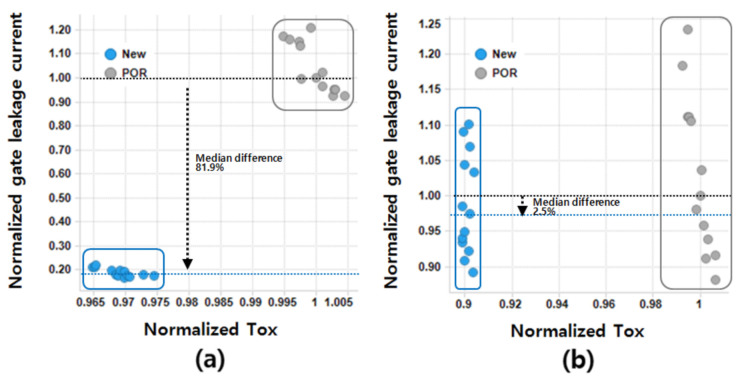
Normalized gate leakage current vs. normalized electrical oxide thickness for (**a**) NFET and (**b**) PFET.

Figure 4 shows the plot of the normalized gate-on-leakage vs. the electrical oxide thickness for the NFET and PFET. The gate leakage current was suppressed by 81.9% for the NFET and 2.5% for the PFET. Note that the gate leakage current was measured in a long-channel MOSFET to avoid short channel effects. The purpose of this was to minimize the impact of the test pattern on leakage current so that the leakage of the film could be accurately measured. Direct numerical comparison of items with different thicknesses is not possible. However, it is noteworthy that the leakage current decreased in spite of the decrease in *T_ox_*. In the case of the NFET (see Figure 4a), the fact that the gate leakage current was not increased (even though *T_ox_* was decreased) is attributed to the change in the threshold voltage (*V_th_*) of the NFET as well as to the increase in the thickness of TiN. Increasing the TiN content has several advantages. In reality, as reported in [13,14], increasing the TiN content can be effective to block boron penetration, thereby reducing interface defects and resistance. It is known that the degree of *V_th_* distribution decreases as the boron penetration decreases (which may be beneficial). The NFET and PFET show different trends because of the fluctuations in *V_fb_* and *V_th_* owing to the change in the thickness of TiN. In addition, the effect of the presence or absence of high-k nitride on the channel is considered to be the key factor in determining the difference between the NFETs and PFETs [15,16,17]. The use of HfSiO as the HK film material, as well as the increase in TiN thickness, also contributes to suppressing leakage. As mentioned in [3], HfSiO is advantageous for suppressing leakage because it has a higher bandgap than that of HfSiON. HfSiON, which is currently in use, has a relatively low bandgap but is used because it was judged to be sufficient for operation. An additional benefit of leakage reduction can be achieved by using HfSiO as the HK material.

**Figure 5:**
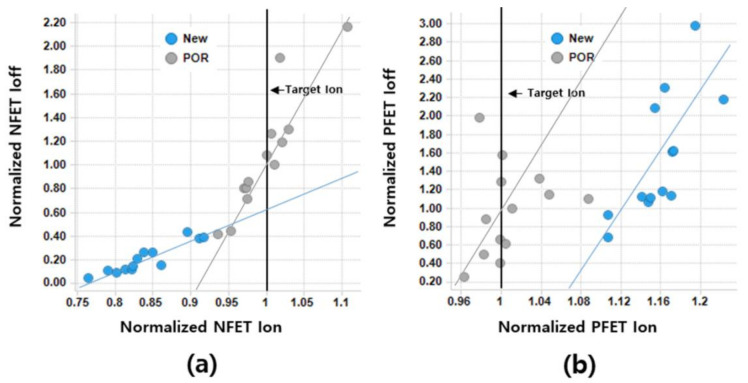
Normalized *I_on_* vs. normalized *I_off_* of (**a**) NFET and (**b**) PFET.

**Figure 6:**
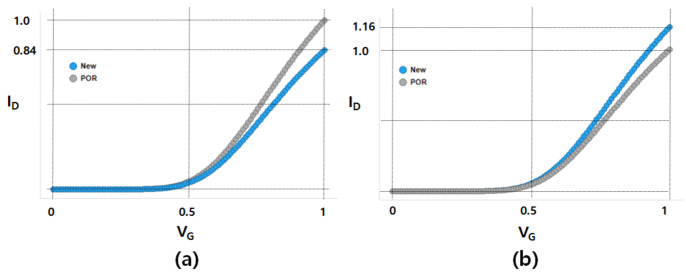
Measured input transfer characteristics of (**a**) NFET and (**b**) PFET.

As shown in Figure 5, the comparison of the on/off current ratio (*I_on_*/*I_off_*) of the POR device against that of the new device (which is fabricated by the newly proposed method) shows that, for the NFET, *I_on_* of the new device (vs. that of the POR device) is decreased by 17.6%, but *I_off_* is decreased by 85.5%. The improvement in *I_on_*/*I_off_* is confirmed more clearly in the NFET than in the PFET. It is noteworthy that *I_on_*/*I_off_* largely differs between the two devices. As the slope of the *I_on_* vs. *I_off_* plot is lower for the new device (see Figure 5a), it follows that *I_off_* of the new device is less increased for a given improvement of *I_on_*. Even if *I_on_* of the new device is enhanced to the level of the POR device’s, the *I_off_* of the new device is expected to be approximately 40% lower than that of the POR device. The slope of the *I_on_* vs. *I_off_* plot for the p-type POR device is almost identical to that for the new p-type device. It is noteworthy that the on/off current ratios for the PFET device are largely comparable to each other. For the PFETs, the *I_on_* of the new device (vs. POR device) is increased by 16%, and *I_off_* is also increased by 18%. Table 2 summarizes the normalized *I_on_*, *I_off_*, and *I_on_/I_off_* of NFETs and PFETs. Although *I_on_*/*I_off_* for PFETs seems to have decreased, it can be considered as an equivalent level within one sigma variation. Note that the PFET’s one sigma of *I_on_*/*I_off_* is 0.35. Figure 6 shows the measured input transfer characteristics. Note that the PFET (vs. the POR device) achieves a higher current for a given gate voltage, but the NFET (vs. the POR device) shows a lower current for a given gate voltage.

Compared to the POR fabrication method, the enhanced device performance achieved with the new fabrication method can be judged to be the main reason for the gain by considering the mobility, as discussed in [7]. The better quality of the HfSiO film leads to better *I_on_*/*I_off_* than that achieved with the HfSiON film if there is no problem with the risk from the heat budget covered in the previous study [18].

Reliability was evaluated at the wafer level, where three evaluations were conducted to prove the quality of the gate oxide and high-k film. The parameters determined include the bias temperature instability (BTI), hot-carrier injury/injection (HCI), and time-dependent dielectric breakdown. These methods are the well-known methods for reliability evaluation, as in [19,20,21,22]. All three evaluation results satisfied the specifications (see Figure 3). As summarized in Table 3, HCI was evaluated based on Δ*ids*, BTI was based on Δ*V_th_*, and TDDB was based on *V_max_*. Using accelerated evaluation (e.g., using a high temperature), all samples are evaluated under the condition that can confirm the lifetime of 10 years (see Table 3). Δ*ids* in HCI evaluation and Δ*V_th_* in BTI evaluation are used to measure the lifetime of the device. Δ*V_th_* is selected based on the value converted from *V_max_*. TDDB also evaluates the reliability of the gate oxide and whether the *V_max_* state can be maintained normally. The reliability evaluation results prove that there is no problem with the heat budget.

## 4. Conclusions

A technique to enhance the on/off current ratio of a 28 nm transistor was proposed and developed. The proposed technique can improve *I_on_*/*I_off_* while keeping the gate leakage current small by adjusting the thickness of TiN. Furthermore, it can enhance the gate-to-channel controllability by lowering the IL. Moreover, it can eliminate the disadvantages of nitride use in the process of using HfSiON as a high-k film. The results of this study prove that the transistor using the HfSiO layer as a high-quality high-k film can afford better *I_on_*/*I_off_* performance than that achieved with the HfSiON film under the limits of maintaining wafer-scale reliability. Further research on how to increase *I_on_*/*I_off_* in PFETs, as well as modifying *V_th_* by adjusting the metal gate and TiN, is necessary to obtain a target *V_th_*. In addition, by diversifying the test pattern size, additional simulations or experiments with various sizes of transistors are required under the improved film quality conditions.

## Figures and Tables

**Table 1:** Geometrical parameters of device structure.

Parameters	NFETs	PFETs
Gate length	28 nm	28 nm
Gate width (normalized)	1*x*	1.4*x*
Gate length(large-scale)	≥1 um	≥1 um
Gate width (large-scale)	≥1 um	≥1 um
Tox	≤2 nm	≤2 nm

**Table 2:** Normalized value for the new device.

Device Parameters	NFETs	PFETs
Normalized *I_on_*	0.824	1.16
Normalized *I_off_*	0.145	1.18
Normalized *I_on_/I_off_*	5.68	0.98

**Table 3:** Detailed criteria for reliability test from JEDEC.

Test Type	Criteria
HCI	Δ*ids* = 10%, 125 °C, AC 10 years
BTI	125 °C, AC 10 years
TDDB	125 °C, AC 10 years
